# Improved Perioperative Seroma and Complication Rates Following the Application of a 2-Layer Negative Pressure Wound Therapy System After Inguinal Lymphadenectomy for Metastatic Cutaneous Melanoma

**DOI:** 10.1245/s10434-020-08513-7

**Published:** 2020-06-05

**Authors:** Marc D. Moncrieff, Riti A. Sharma, Esther Gathura, Martin J. Heaton

**Affiliations:** 1 Department of Plastic and Reconstructive Surgery, Norfolk and Norwich University Hospitals NHS Foundation Trust, Norwich, UK; 2 Norwich Medical School (University of East Anglia), Norwich, UK

## Abstract

**Background:**

Perioperative complications following inguinal lymphadenectomy, including seroma formation, are frequent. We have employed a 2-layer negative pressure wound therapy (2-LNPWT) as a method to reduce seroma rate and perioperative complications. We present the outcome of our initial experience with 2-LNPWT and compare the outcomes of its use with traditional closed suction drains (CSDs).

**Materials and methods:**

A non-randomised retrospective case–control series was analysed. Surgeons performing inguinal lymphadenectomy for metastatic cutaneous melanoma utilised either the 2-LNPWT therapy or traditional CSDs according to their practice preference.

**Results:**

The study included 111 patients. The cohorts were well matched for gender, disease burden, body mass index and comorbidities. The 2-LNPWT technique was associated with significantly better postoperative outcomes than CSD, in terms of incidence of seroma formation (26.9% vs 49.4%; *p* < 0.03), period of drainage (15 days vs 20 days; *p* = 0.005) and return to theatre rate (0% vs 15.3%; *p* = 0.03). The overall seroma rate was 44.1%. The only significant association with seroma initiation was the type of drainage system used (2-LNPWT 31.2% vs CSD 58.3%; *p* < 0.03; OR 3.0). The method of drainage did not alter the course of an established seroma. There was no significant difference in overall or disease-specific survival detected between the 2 groups.

**Conclusion:**

This retrospective non-randomised case control study has demonstrated the safe use of a novel application of negative pressure wound therapy that significantly reduced the incidence of seroma formation and postoperative complication rate for inguinal lymphadenectomy for melanoma.

Inguinal lymphadenectomy is the current standard of care in the management of macroscopic lymph node metastasis. Postoperative complications following inguinal dissection commonly include seromas, wound infection, impaired wound healing, cellulitis and skin necrosis.^1^,^2^ Seroma formation accounts for 32–80% of presentations postoperatively.^2^ These patients routinely return to outpatient clinics for aspiration and resolution of the problem is often protracted. As a direct result of the repeated seroma aspirations and drainage, subsequent complications such as infection may follow. This often leads to unscheduled returns to theatre for a further surgical procedure and in turn could result in suboptimal rehabilitation and potential disability.

Negative pressure wound therapy (NPWT) was originally indicated for complex wounds to promote healing. Its use has become widespread in reconstructive, oncological and emergency surgery to initiate wound healing, to reduce infection rates and to stabilise challenging wounds.^3^–^7^ The underlying mechanism of action is to increase the vascularity of the wound bed by promoting granulation tissue formation. The direct action of the negative pressure within the dressing also serves to remove excess fluid and reduce wound oedema. More recently, the indications for NPWT have expanded to include the management of high-output exudate and seromas by employing it as an alternative method to closed suction draining.^8^

In our unit, a modified two-layer negative pressure wound therapy (2-LNPWT) dressing in combination with a standard negative pressure dressing system [Renasys Go, Smith & Nephew, UK] has routinely been employed as a standard of care to manage inguinal lymphadenectomy wounds postoperatively by the senior author (MM). The rationale for employing the 2-LNPWT technique is that it acts as a traditional closed-suction drainage (CSD) system whilst simultaneously applying continuous external compression to the skin and the wound cavity deep to it. The CSD was employed as a standard of care by the other senior author (MH). We undertook a retrospective non-randomised case–control study over a 4-year period to investigate whether there was any evidence for a perioperative therapeutic advantage of 2-LNPWT over traditional closed suction drainage.

## Methods and Materials

This case-series review was registered as an audit at the Norfolk & Norwich University Hospital (Audit Reference Number: Plas-15-16-003, registered November 2015). All patients were treated at a single tertiary-referral university hospital cancer centre and the patients were identified from our prospectively maintained melanoma database. Further surgical and follow-up data were obtained from the patients’ electronic records and case written notes. Missing data held by other centres were requested and completed wherever achievable. Data were collected on patient demographics and diagnosis, in addition to admission and readmission data, seroma formation rates, total number of days with drain, return to theatre rate and other standardised complications as collected for our monthly morbidity and mortality meetings. Examples of the latter included postoperative wound infection, haematoma and wound dehiscence. Disease-specific and overall survival outcomes were obtained from the prospective and contemporaneous melanoma database.

Statistical collection was performed using Excel. Non-parametric statistical tests such as Fisher’s exact test and the Mann–Whitney *U* test were utilised and an alpha of 0.05 was used as the cut-off to determine statistical significance. For the purpose of this study, a seroma was defined as a symptomatic postoperative collection of lymphatic fluid at the lymphadenectomy site, following removal of the drain, that required an intervention (usually aspiration) to remove or reduce it. A perioperative complication was defined as any untoward event that started within 31 days of lymphadenectomy. The Clavien Dindo system^9^ was used to classify complication severity. Lymphoedema rates were specifically not measured in this study, due to the unpredictable onset postoperatively of the condition.

## Operative Technique

Patients were identified as being suitable for lymphadenectomy and the procedure was performed after discussion within the multidisciplinary team meeting. The patients were assessed and appropriately counselled by their surgeon, prior to embarking on surgery. The operative technique consisted of standardised open lymphadenectomy which included an oblique elliptical incision extending from the groin crease infero-medially to the apex of the femoral triangle. The extent of the skin excision was arbitrarily determined intra-operatively by the surgeon depending on the available laxity of the skin overlying the femoral triangle. Skin flaps were raised at the level of the superficial fascia to the extent of the boundaries of the femoral triangle. Inguinal lymph nodes were removed en bloc, incorporating the long saphenous vein in the specimen. Large, visible lymphatics were ligated en masse with vicryl sutures at the proximal and distal limits of the femoral triangle. A sartorius muscle turn-over flap was not performed.

**Fig. 1:**
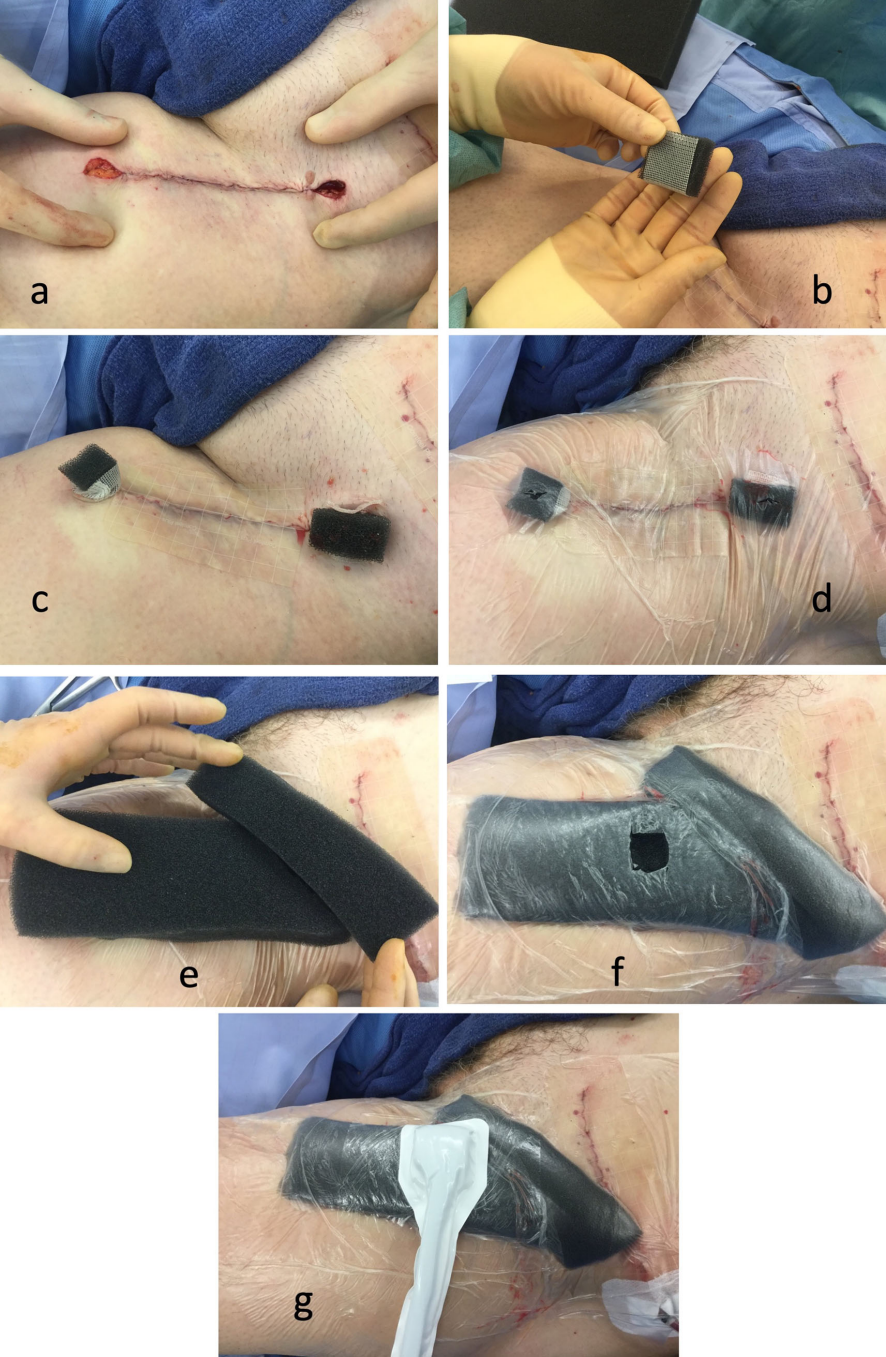
Stepwise demonstration of application of 2-LNPWT dressing after inguinal dissection.** a** Wound closure leaving distal and proximal ends open approx 1–2 cm.** b** Foam wick with silicone dressing to prevent granulation tissue formation.** c** Wicks inserted in each wound opening.** d** Adherent film applied over wound to create a seal and perforated over each foam wick.** e** 2nd layer of foam cut to the dimensions of the dissection cavity.** f** 2nd layer of adherent film over foam and perforated in centre.** g** Connection tubing attached over perforation running laterally to pump (Note: separate incison in left iliac fossa for pelvic dissection)

A 2-LNPWT system was chosen by one surgeon (MM) on the basis of the perceived ability of the patient to be able to safely mobilise with the dressing and access outpatient care readily in the postoperative period. Where these 2 criteria were not met in the opinion of that surgeon, CSD was used instead. The other surgeon (MH) utilised only CSD. Where a CSD system was employed, a drain was inserted prior to wound closure (15 Fr Blake drain: Ethicon Inc, Johnson & Johnson) exiting in the vicinity of the anterolateral mid-thigh where it was firmly secured to the patient with sutures before connecting to a vacuum drainage bottle. The sequence of images in Fig. 1 demonstrates the deployment of the alternative 2-LNPWT dressing. Essentially, the wound is sutured in layers, save for the terminal 1–2 cm at each end (Fig. 1a). Foam wicks are inserted into the residual defect and covered with a non-adherent silicone dressing, which prevents premature closure and excess granulation tissue formation at the exit sites (Fig. 1b–d). An occlusive dressing is placed over the wound and is perforated over the foam wicks. A second, separate layer of foam, wide enough to cover the extent of the underlying dissection cavity, is placed over the wound and this is sealed with a second occlusive dressing (Fig. 1e). The occlusive dressing is then perforated (Fig. 1f) to allow placement of the connecting tubing (Fig. 1g) and connected to the suction unit. The unit was invariably set to 75 mmHg of continuous negative pressure.

Postoperatively, patients are mobilised the day after surgery and are discharged home the following day. The standard protocol for the management of the CSD system was to continue drainage until the daily output total was less than 30 ml in a 24-h period. CSDs were taken off suction at 5 days and continued with ‘free’ gravitational drainage until removal. The philosophy behind this protocol is to prevent propagation of the lymphorrhoea at the drain site by the negative pressure in the cavity. In the case of the 2-LNPWT system, the entire dressings were changed every 3–4 days by the plastic surgery nurses in the dressings clinic until the canister demonstrated little or no drainage over a 24-h period. This was a clinical judgement based upon the volume of altered silica gel compound in the canister at review. In all cases, the drainage period recorded was the number of days calculated from the date of the surgery to the date of removal of the drainage system.

## Results

A total of 133 patients were identified retrospectively over a 5-year period between January 2010 and November 2015. Complete datasets were available for analysis on 111 patients (83.5% of cohort). There were 39 males and 72 females (35.1% vs 64.9%). The median age was 66.0 years [IQR 56.0–75.0]. There were 40 smokers (36.0%) and the median BMI was 26.1. Fifty patients (45.0%) underwent surgery for a positive sentinel biopsy compared to 61 (55.0%) who underwent a therapeutic procedure for palpable disease. Approximately half the patients (51.3%) underwent a simultaneous pelvic dissection. The median N-ratio was 0.17 [IQR 0.10–0.27] and extracapsular spread was identified in the resection specimen in over a fifth of patients (22.5%). The overall return to theatre rate was 11.7% (13 patients). Postoperatively the drains remained in situ for a median of 17 days [IQR 12.8–25.0 days]. Seroma data were missing for 17 patients (15.3%) but the overall incidence of seroma formation was 52.9% (49/94 patients). The median total seroma volume was 620 ml. The overall complication rate was 35.1% (39 participants), though the majority of these were trivial, Grade I episodes (59.0%).

Table 1 outlines the comparison of patient demographics, tumour characteristics and burden, pathology and perioperative course between the 2-LNPWT and CSD groups. The patients in the 2-LNPWT were significantly younger (57.5 vs 69.0 years; *p* < 0.007), otherwise the groups were well-matched for gender, ASA grade, smoking status and BMI. Similarly, both groups were well-matched for burden of disease, indications for surgery, extent of surgery and risk for regional recurrence (extracapsular spread). Whilst there was no significant difference in complication rate, severity of complications or seroma volume drained between the two groups, there was a significantly reduced seroma rate (29.6% vs 49.4%; *p* < 0.03) and a significantly reduced return to theatre rate (0% vs 15.3%; *p* = 0.03) in the 2-LNPWT group. The median period of drainage was significantly reduced by almost 1 week in the 2-LNPWT group (15 vs 20 days; *p* = 0.005) compared to the CSD group. There was no significant difference in overall or disease-specific survival detected between the 2 groups. The median survival overall and disease-specific survival was 37 months and 56 months, respectively in the CSD group, and not reached in the 2-LNPWT group. The median disease-free interval was 16.5 months in the 2-LNPWT group and 30.5 months in the CSD group. This disease-free survival difference did not reach statistical significance (*p* = 0.1) and multivariate analysis with Cox’s proportional hazards method identified a number of positive nodes as the only significant independent predictor of relapse (*p* = 0.01; HR = 1.17 [1.07–1.28]). There were only two instances of in-field nodal relapse, both in the CSD group.

**Table 1:** Comparison of patient demographics, tumour characteristics and burden, pathology and perioperative course between 2-LNPWT and CSD groups

Variable	2-LNPWT *n* = 26 (%)	CSD *n* = 85 (%)	Total	Significance
Demographics				
Gender				
Male	6 (23.0)	33 (38.8)	39 (35.1)	ns
Female	20 (76.9)	52 (61.2)	72 (64.9)	
Age				
Median	57.5	69.0	66	*p *< 0.007
IQR	[46.8–67.8]	[59.0–76]	[56.0–75.0]	
ASA grade				
0	2 (7.7)	6 (7.0)	8 (7.2)	ns
1	5 (19.2)	14 (16.5)	19 (17.1)	
2	15 (57.7)	53 (62.4)	68 (61.3)	
3	4 (15.4)	12 (14.1)	16 (14.4)	
Smoker				
Yes	7 (26.9)	33 (38.8)	40 (36.0)	ns
No	19 (73.0)	52 (61.2)	71 (64.0)	
BMI				
Median	27.0	25.8	26.1	ns
IQR	[25.0–30.8]	[23.8–29.6]	[24.2–30.0]	
Tumour burden and pathology				
Indication for surgery				
Completion lymph node dissection	16 (61.5)	34 (40.0)	50 (45.0)	ns
Therapeutic	10 (38.5)	51 (60.0)	61(55.0)	
Pelvic dissection				
Yes	9 (34.6)	48 (56.5)	57 (51.3)	ns
No	17 (65.4)	37 (43.5)	54 (48.7)	
N-ratio				
Median	0.13	0.17	0.15	ns
IQR	[0.11–0.2]	[0.09–0.29]	[0.10–0.27]	
Extracapsular spread				
Absent	15 (57.7)	37 (43.5)	52 (46.8)	ns
Present	4 (15.4)	21 (24.7)	25 (22.5)	
Not stated	7 (26.9)	27 (31.8)	34 (30.6)	
Perioperative course				
Length of stay (days)				
Median	8	8	8	ns
IQR	[5.3–10.5]	[6.0–11.0]	[6.0–11.0]	
Return to theatre				
No	26 (100.0)	72 (84.7)	98 (88.3)	*p *= 0.03
Yes	0 (0.0)	13 (15.3)	13 (11.7)	OR: n/a
*Haematoma*			*2*	
*Wound dehiscence*			*5*	
*Wound infection*			*6*	
Seroma				
No	15 (57.7)	30 (35.3)	45 (40.5)	*p *< 0.03
Yes	7 (26.9)	42 (49.4)	49 (44.1)	OR: 3.0
Not stated	4 (15.4)	13 (15.3)	17 (15.3)	(1.1-8.3)
Period with drain (days)				
Median	15	20	17	*p *= 0.005
IQR	[9.5–15.8]	[15.8–26.3]	[12.8–25.0]	
Seroma aspirations (n)				
Median	1	0	0	ns
IQR	[0–3]	[0–3]	[0–3]	
Total seroma volume (ml)				
Median	525	690	620	ns
Complication (n)				
No	19 (73.0)	53 (62.3)	72 (64.9)	ns
Yes	7 (26.9)	32 (37.6)	39 (35.1)	
Complication subtype				
Haematoma	0	2 (6.3)	2 (5.1)	
Thromboembolism	1 (14.3)	0	1 (2.6)	
Post-op confusion	0	1 (3.1)	1 (2.6)	
Neuropraxia	1 (14.3)	0	1 (2.6)	
Wound dehiscence	0	7 (21.9)	7 (17.9)	ns
Wound infection	5 (71.4)	22 (68.8)	27 (69.2)	
Complication severity^a^				
0	19 (73.0)	53 (62.4)	72 (64.9)	ns
I	5 (19.2)	18 (21.2)	23 (20.7)	
II	1 (3.8)	2 (2.4)	3 (2.7)	
IIIA	1 (3.8)	1 (1.1)	2 (2.7)	
IIIB	0 (0.0)	11 (12.9)	11 (9.9)	
Survival (median, months)				
Overall survival	Not reached	37	38	ns
Disease-specific survival	Not reached	56	56	ns

^a^The Clavien–Dindo^9^ classification of surgical complications

Table 2 outlines the comparison between the groups of patients who developed a seroma (*n* = 49, 52.9%) and those who did not (*n* = 45, 47.9%). There were no significant risk factors identified for the development of seroma in terms of patient demographics, comorbidities or tumour burden. Similarly, tumour burden, extent of surgery, and complication rate were not associated with seroma formation. CSD was associated with a significantly increased risk of seroma formation in our cohort (31.2% vs 58.3%; odds ratio = 3.0 [95% CIs 1.1–8.3], *p* < 0.03). The method of drainage used was the only significant risk factor associated with the development of postoperative seroma identified in our cohort.

**Table 2:** Comparison of patient demographics, tumour characteristics and burden, pathology and perioperative course between the seroma formation and no seroma formation groups

Variable	No seroma formation *n* = 45 (%)	Seroma formation *n* = 49 (%)	Total	Significance
Demographics				
Gender				
Male	16 (35.6)	19 (38.8)	35 (37.2)	ns
Female	29 (64.4)	30 (61.2)	59 (62.8)	
Age				
Median	65.0	66.0	65.5	ns
IQR	[53–74]	[57–76]	[56.0–75.8]	
ASA grade				
0	3 (6.7)	5 (10.2)	8 (8.5)	ns
1	7 (15.6)	9 (18.4)	16 (17.0)	
2	27 (60.0)	30 (61.2)	57 (60.6)	
3	8 (17.8)	5 (10.2)	13 (13.8)	
Smoker				
Yes	25 (55.6)	30 (61.2)	55 (58.5)	ns
No	20 (21.3)	19 (38.8)	39 (41.5)	
BMI				
Median	25.7	26.3	26.1	ns
IQR	[23.8–28.6]	[24.5–29.2]	[24.1–28.8]	
Tumour burden and pathology				
Indication for surgery				
Completion lymph node dissection	19 (42.2)	20 (40.8)	39 (41.4)	ns
Therapeutic	26 (57.8)	29 (59.2)	55 (58.5)	
Pelvic dissection				
Yes	23 (51.1)	24 (49.0)	47 (50.0)	ns
No	22 (48.9)	25 (51.0)	47 (50.0)	
N-ratio				
Median	0.20	0.16	0.17	ns
IQR	[0.09–0.29]	[0.11–0.20]	[0.10–0.27]	
Extracapsular spread				
Absent	23 (51.1)	21 (42.9)	44 (46.8)	
Present	10 (22.2)	11 (22.4)	21 (22.3)	ns
Not stated	12 (26.7)	17 (34.7)	29 (30.9)	
Perioperative course				
Length of stay (days)				
Median	8	8	8	ns
IQR	[5.3–10.5]	[6–11]	[6–11]	
Return to theatre				
No	40 (88.9)	43 (87.8)	83 (88.3)	ns
Yes	5 (11.1)	6 (12.2)	11 (11.7)	
Complication (n)				
No	33 (73.3)	27 (55.1)	60 (63.8)	ns
Yes	12 (26.7)	22 (44.9)	34 (36.1)	
Complication severity^a^				
0	33 (35.1)	27 (28.7)	60 (63.8)	ns
I	7 (7.4)	16 (17.0)	23 (24.5)	
II	2 (2.1)	1 (1.1)	3 (3.2)	
IIIA	0 (0.0)	0 (0.0)	0 (0.0)	
IIIB	3 (3.2)	5 (4.3)	8 (8.5)	
Drainage system				
2-LNPWT	15 (68.8)	7 (31.2)	22	*p *< 0.03
CSD	30 (41.7)	42 (58.3)	72	OR 3.0 (1.1–8.3)
Total	45 (47.9)	49 (52.1)	94	
Period with drain (days)				
Median	17	18	17	ns
IQR	[9.5–15.8]	[15.8–26.3]	[15.0–25.0]	

^a^The Clavien–Dindo^9^ classification of surgical complications

## Discussion

It is generally acknowledged that inguinal lymphadenectomy is a standard procedure in the surgical oncology armamentarium that has a disproportionately high complication rate.^2^ Whilst the complications are generally non-life threatening and localised to the site of operation, they are often protracted and have a substantial impact on the patients’ quality of life. One of the most common postoperative complications in the perioperative period is seroma.^1^,^10^,^11^ There have been multiple studies that have investigated interventions designed to either reduce the incidence or reduce the extent of the problem once it has become established.^12^–^15^ A recent systematic review by the Cochrane Group^11^ identified a lack of any useful data and concluded that, “…there is a need for high quality RCTs to guide clinical practice in this under-researched area.” Unfortunately, there have been no studies to date that have demonstrated a reliable method to reduce the incidence of seroma.

This retrospective cohort study has attempted to compare both the incidence of postoperative seroma formation and the duration of seroma drainage following inguinal lymphadenectomy for metastatic melanoma between two methods of wound drainage. Whilst there was no randomisation of the cohort between the two systems, the cohorts were well matched in terms of demographics, disease burden and extent of surgery. There was a significant bias towards younger patients for the 2-LNPWT system, which probably reflects the perceived ability of the patient by the treating clinician to manage the bulkier vacuum pump system and/or the opportunity to return to the outpatient department to have the system changed by the clinic nurses.

The key finding of this study was that there was a significant association with better postoperative outcomes (Table 1) using the 2-LNPWPT system in terms of incidence of seroma formation (26.9% vs 49.4%; OR 3.0; *p* < 0.03), period of drainage (15 days vs 20 days; *p* = 0.005) and return to theatre rate (0% vs 15.3%; *p* = 0.03). The authors hypothesise that the dual action of the 2-LNPWT may be the reason for these findings. The 2-LNPWT system is designed to act both as a standard closed suction drainage system and a simultaneous compression dressing. We hypothesise that the key step in preventing the seroma is the promotion of closure and healing of the soft tissue dead space between the flaps of skin with subcutaneous fat and the floor of the dissected femoral triangle. Theoretically, the compression element acts at a pressure greater than the occlusion pressure of the afferent lymphatic vessels in a uniform manner over the operated area, thereby preventing the flow of lymph through the transected vessels into the cavity. In addition the drain removes any lymphatic fluid that would otherwise accumulate in the cavity. Previous studies have shown that compression dressings alone^16^ do not change the incidence of seroma formation or the duration of seroma drainage. In contrast, the clinical impact of our system was significant. The period of postoperative drainage was reduced by almost 1 week and the incidence of seromas was halved. The substantial decrease in the return to theatre rate was due to the reduced incidence of infected seromas arising in turn from a reduction in the need for repeated needle aspirations in the outpatient clinic.

The overall incidence of postoperative seroma was 52.9%, which was consistent with previously published data.^2^ When the factors for seroma formation were analysed (Table 2), the only significant association was the type of drainage system used (2-LNPWT 31.2% vs CSD 58.3%; *p *< 0.03; OR 3.0 [1.1–8.3]). Unlike previous studies,^16^ there was no association with body mass index, age, gender, ASA grade or smoking status. Interestingly, the data in Table 1 shows no difference in the number of seroma aspirations or mean seroma volume between the two drainage systems. This data would suggest that the optimal management of a seroma is to prevent it happening at all. Unfortunately, the duration of seroma formation (from clinical identification to clinical resolution) was not consistently recorded in our dataset, though it is clear from Table 1 that, once a seroma was established, it was not possible to reliably alter the clinical course of this complication, regardless of the initial drainage system that was employed postoperatively. These findings are consistent with previous studies. Of note, one study found that the use of sclerosants to reduce the clinical course of postoperative seromas paradoxically increased the duration of the drainage period.^16^ One conclusion that could be drawn from this data is that the outcome of seroma formation should, perhaps, be regarded as a common sequela to the procedure rather than a complication. According to Dindo and Clavien,^17^ a complication is defined as, “…any deviation from the normal postoperative course,” whereas a sequela is defined as, “…an after-effect” of surgery that is inherent to the procedure. The data in this study shows that incidence of seroma formation, though common, can be modified by postoperative interventions, such as 2-LNPWT. Encouragingly, the data also shows that a seroma was not associated with a significant increase in other complications, when managed at our unit.

**Fig. 2:**
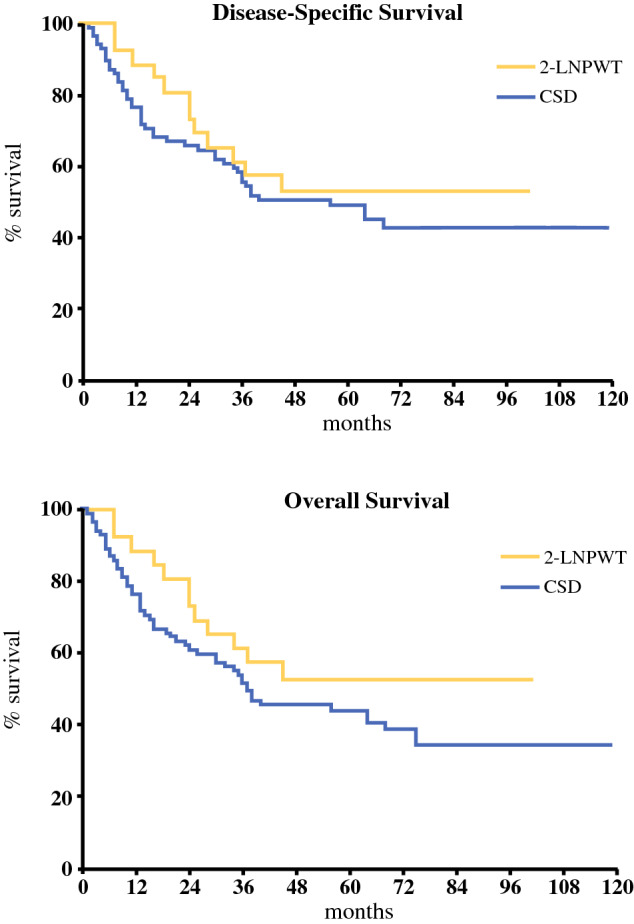
Kaplan–Meier curves showing disease-specific and overall survival between the CSD and 2-LNPWT groups

The use of vacuum-assisted closure therapy is generally considered a contraindication for wounds in the presence of malignancy because there are theoretical concerns that it may promote tumorigenesis and secondary metastasis by the induction of angiogenesis in the wound bed.^18^ When the disease outcomes were analysed for this cohort, the data demonstrated that the use of the 2-LNPWT is not associated with a worse disease-specific or overall survival, which would suggest that it is safe to use in patients after melanoma lymphadenectomy. Indeed, Fig. 2 demonstrates a (non-significant) improved survival in favour of the 2-LNPWT, though this is highly likely to be due to the significant younger age bias in this cohort (Table 1), rather than any direct benefit from the drainage system per se. The design of the 2-LNPWT included a non-adhesive dressing around the foam wick inserted into the wound, which was a deliberate ploy to prevent granulation tissue and premature wound closure at the drainage apertures. Accordingly, the theoretical risk of promoting tumorigenesis through neo-angiogenesis was reduced to a minimum. Similar data is emerging that suggests that the use of negative pressure wound therapy in other tumour sites can be safely achieved in carefully selected patients.^18^–^20^

There are potential limitations and biases in this study that merit comment. First, there was a lack of randomisation of drainage systems applied to the patients, in addition to a lack of randomisation of drainage techniques applied by the two surgeons in this study. Whilst the proportion of patients undergoing completion lymph node dissection was greater in the 2-LNPWT (61.5% vs 40.0%) and the proportion of pelvic dissections was less (34.6% vs 56.5%), neither differences were statistically significant and, overall, the similarity of the case-mix between the two cohorts (Table 1) suggests a lack of bias in this regard. It is possible that the significant outcome differences observed may be due to surgical technique, though this is unlikely given that the surgeons use the same technique to perform inguinal lymphadenectomy and had similar training (both in the UK & Australia). Second, patients in the CSD group did not routinely attend outpatient clinics in a prescribed manner when compared to the 2-LNPWT cohort, who required a visit every 3–4 days to replace the complex dressing. Furthermore, the oversight of the drain removal in the CSD cohort was delegated to the plastic surgery nursing staff and the duty resident in the department. The reasons for drain removal were not always recorded and some drains may have been removed prematurely for fear of ascending infection that may ultimately have been unfounded. These potential biases may account in some way for the differences in complications and seroma rates observed between the two groups. Third, we did not investigate the incidence of lymphoedema in this cohort, therefore we cannot comment on the association of the two systems with this particular outcome. However, studies have shown an associated increase in lymphoedema with postoperative surgical site infection.^10^,^15^ It is advocated that any future randomised study should include lymphoedema as an endpoint. Finally, it is worth commenting that the median length of stay was 8 days for both cohorts. This could be considered excessive in a modern surgical practice and the authors would not disagree with this contention. The protocol for the management of the drains and the hospital discharge was changed mid-way through the audit cycle and it is now uncommon for patients to remain as an in-patient beyond 2 days in our service, regardless of the drainage system used. Accordingly, it is unlikely that this contributed to any biases in this study.

## Conclusion

This retrospective non-randomised case control study has demonstrated the safe use of a novel application of negative pressure wound therapy that substantially improves the incidence of seroma formation and postoperative complication rate for inguinal lymphadenectomy for melanoma. This data could possibly be extrapolated for other disease indications requiring inguinal lymphadenectomy, though we would advise caution. However, given the encouraging data we would recommend that a prospective phase III RCT is undertaken to validate our findings. Similarly, we would also encourage the commercial sector to invest in product design to improve the facility for applying and changing the dressings in both the hospital and primary care settings.
